# Optimization and identification of siderophores produced by *Pseudomonas monteilii* strain MN759447 and its antagonism toward fungi associated with mortality in *Dalbergia sissoo* plantation forests

**DOI:** 10.3389/fpls.2022.984522

**Published:** 2022-11-07

**Authors:** Pragati Srivastava, Manvika Sahgal, Khanchand Sharma, Hesham Ali El Enshasy, Abdul Gafur, Saleh Alfarraj, Mohammad Javed Ansari, R. Z. Sayyed

**Affiliations:** ^1^Department of Microbiology, G. B. Pant University of Agriculture and Technology, Pantnagar, Uttarakhand, India; ^2^Department of Agricultural Engineering, Central University of Nagaland, School of Agricultural Sciences and Rural Development, Dimapur, India; ^3^Institute of Bioproduct Development (IBD), UniversitiTeknologi Malaysia (UTM), Skudai, Malaysia; ^4^School of Chemical and Energy Engineering, UniversitiTeknologi Malaysia (UTM), Skudai, Malaysia; ^5^Institute of Bioproduct Development (IBD), City of Scientific Research and Technology Applications (SRTA), Alexandria, Egypt; ^6^Sinarmas Forestry Corporate Research and Development, Perawang, Indonesia; ^7^Zoology Department, College of Science, King Saud University, Riyadh, Saudi Arabia; ^8^Department of Botany, Hindu College, Moradabad (Mahatma Jyotiba Phule Rohilkhand University, Bareilly), Moradabad, India; ^9^Asian PGPR Society, Department of Entomology, Auburn University, Auburn, AL, United States; ^10^Department of Microbiology, PSGVP Mandal's S. I. Patil Arts, G. B. Patel Science and STKV Sangh Commerce College, Shahada, India

**Keywords:** siderophore, *Pseudomonas*, optimization, antagonism, Fe concentration

## Abstract

Siderophore-positive bacteria present in the rhizosphere and in bulk soil assist plants by either inhibiting phytopathogen proliferation or increasing plant growth. The bacterial diversity of the Shisham forest ecosystem in the Tarai region of the Western Himalayas was studied and used for siderophore production, taking into account the large-scale dieback and wilt-induced mortality in *Dalbergia sissoo* (common name: shisham) plantation forests and the importance of soil microbes in tree health. In addition*, Pseudomonas, Burkholderia*, and *Streptomyces* were prominent siderophore-positive bacteria in Shisham forests. *Pseudomonas* species are known for their remarkable siderophore-producing ability. Bacterial siderophores inhibit pathogen growth by rapidly lowering the number of ferric ions in the rhizosphere. The *Pseudomonas monteilii* strain MN759447 was isolated from a *D. sissoo* plantation forest at the Agroforestry Research Centre, Pantnagar, Uttarakhand (28°58′N 79°25′E/28.97°N 79.41°E). It produces a significant number of siderophore units (80.36% in total). A two-stage optimization of growth factors was attempted in the strain MN759447 for better siderophore recovery. In the first-stage single-factor experiment, among the five variables studied, only pH, NH_4_NO_3_ concentration, and Fe concentration affected siderophore synthesis. In the second stage, an optimization of pH, NH_4_NO_3_ concentration, and Fe concentration for improved growth and enhanced siderophore production was carried out using a Box–Behnken design with response surface methodology. By using LC-MS, two derivatives of pseudomonine, salicylic acid, and kynurenic acid were detected as siderophores in the purified XAD-2 methanol extract of the *P. monteilii* strain MN759447. In addition to siderophore production, the *P. monteilii* strain MN759447 also exhibited a broad range of antagonistic activity against *Aspergillus calidoustus* (65%), *Fusarium oxysporum* (41.66%), *Talaromyces pinophilus* (65%), and *Talaromyces verruculosus* (65.1%) that are linked to sissoo mortality. To our knowledge, this is the first report on siderophore-producing bacteria isolated, identified, and characterized from the *D. sissoo* Roxb. forest habitat. This strain can also be developed as a commercial product.

## Introduction

Premium-quality timber trees are becoming extinct because of a high mortality rate due to infection caused by fungal species that induce symptoms of dieback and wilt in the tree ecosystem. *Cercospora sissoo, Fusarium solani, Ganoderma lucidum, Phyllactinia dalbergiae, Phellinus dalbergiae*, and *Phytophthora cinnamomi* are a few fungal species that have been reported to reduce the yield and production of *D. sissoo* timber trees (Naqvi et al., 2019; Ghazali et al., 2022). The pathogenic fungi manifest as the dieback of branches, bark splitting, and main stem gummosis, leading to the death of a tree. In addition to these, symptoms like wilting, cankers, internal chlorosis, and necrosis may also appear after infection (Javaid, 2008; Rehman et al., 2012). Plant growth-promoting bacteria possessing biofungicide potential are residents of the rhizosphere (Qessaoui et al., 2022). The key species among them belong to *Pseudomonas*, a genus that has phytopathogen biocontrol ability (Keerthana et al., 2022). The microbial population assemblage within the rhizosphere chiefly depends on the soil physicochemical properties and plant exudates in the soil (Khan et al., 2021). Plants differing in genotypes are also responsible for the promising microbiome selection (Jamil et al., 2022). Recently, Joshi et al. (2021) demonstrated contrasting bacterial communities in healthy and wilted *D. sissoo* Roxb. forests. A metagenomic analysis showed a lower accumulation of beneficial phyla such as proteobacteria but an abundance of pathogenic genera such as *Brevibacterium, Blastocatella, Methylobacterium*, and *Williamsia* in the wilted forest. Hence, there is an immense need to identify the contribution of rhizosphere microbiota to plant iron nutrition. Notably, a study conducted by Lurthy et al. (2020) reported an abundance of protein families that are directly linked to siderophore synthesis in bacterial communities, majorly *Pseudomonas*. Also, *Acetobacter, Acinetobacter, Alcaligenes, Arthrobacter, Azoarcus, Azospirillum, Azotobacter, Bacillus, Beijerinckia, Burkholderia, Derxia, Enterobacter, Gluconacetobacter, Herbaspirillum, Klebsiella, Ochrobactrum, Pantoea, Pseudomonas, Rhodococcus, Serratia, Stenotrophomonas*, and *Zoogloea* were reported to confer beneficial impacts on plant growth and development (Alawiye and Babalola, 2019). Rhizosphere microbiota exert beneficial effects on plants through several direct and indirect mechanisms. The mechanisms that exert a direct beneficial reaction on plants are phytohormones, siderophores, antioxidants, cell wall-degrading enzymes, and VOCs. In addition to this, the solubilization of minerals (e.g., phosphorus, potassium, and zinc) and biological nitrogen fixation also provide direct benefits. Indirect benefits are provided through antibiosis, induced systemic resistance, and biofilm formation (Singh et al., 2022). Siderophore-producing PGPRs are also involved in pathogen biocontrol *via* competition for iron (Fe^3+^) (Murthy et al., 2021). Siderophores are low-molecular weight (400–2000 Da) complex protein chelators having a high affinity for ferric iron (Neilands, 1995; Chen et al., 2004; Ahmed and andHolmström, 2014). They are secreted under iron-deficient conditions to scavenge iron from insoluble iron (III) complexes. Iron accounts for up to 5% of the Earth's crust and is present in all soil types. Ferromagnesian silicates form a significant portion of iron in the soil, which is converted into weak water-soluble iron oxides upon weathering (Schwertmann, 1991). Hence, the bioavailability of Fe in the soil is very limited in contrast to the total Fe concentration. Inorganic forms of iron such as Fe^3+^, Fe (OH)^2+^, and Fe^2+^ are present at low concentrations in the soil. In the aerobic environment, as Fe^2+^ is scarce, Fe^3+^ must be taken up by the bacteria to maintain iron homeostasis within the cell. In our previous study (Srivastava et al., 2020), the soil Fe content in the *D. sissoo* plantation forest was found to be between 19.53 kg ha^−1^ (RS) and 17.46 kg ha^−1^ (BS) in the monsoon season and between 17.45 kg ha^−1^ (RS) and 12.58 kg ha^−1^ (BS) in the cold dry season, which are below the threshold of 40,000 kg ha^−1^ for 50 cm of agricultural land (Shenker and Chen, 2005). Thus, the bioavailable form of iron was restricted to set the stage for siderophore-producing bacteria to overcome Fe deficiency. While iron-deficient conditions exert a positive influence on siderophore production, other abiotic factors such as carbon source, nitrogen source, pH, temperature, and heavy metals are the major factors governing siderophore synthesis (Kumar et al., 2017; Yu et al., 2017). pH plays a very sensitive role in siderophore synthesis. It determines the solubility and availability of iron for organism growth. According to a study, neutral pH is suitable for siderophore production in S-11 (Tailor and Joshi, 2012). Also, carbon and nitrogen sources are important for siderophore production. Maltose as a C source and NH_4_NO_3_ as an N source significantly enhance siderophore production in endophytic fungi from *Cymbidium aloifolium* (Chowdappa et al., 2020). The presence of metal ions in the medium affects the production of siderophores. Dimkpa et al. (2009), Sayyed and Chincholkar (2010) reported that the Cd metal influenced siderophore production in *Streptomyces tendae* (F4). Different bacteria and fungi produce “siderophores” that are optimized for Fe^3+^ binding. Based on their iron-binding moieties, siderophores can be classified into three basic types: catecholate, hydroxymate, and carboxylate. In addition, there is a fourth type that contains more than one iron-binding moiety (Raymond et al., 2003). Siderophore-producing bacteria have immense importance in crop nutrition and phytopathogen suppression (Sayyed et al., 2010, 2013). They colonize the root rhizosphere of several crops and secrete various antifungal metabolites (Haas and Défago, 2005). Ghazy and El-Nahrawy (2021) studied siderophore production in *Bacillus subtilis* MF497446 and *Pseudomonas koreensis* MG209738 and also evaluated their efficacy in controlling *Cephalosporium maydis* in maize. Siderophore-producing *P. putida* suppresses *Fusarium* wilt pathogens of cucumber, radish, and flax, demonstrating pathogen biocontrol by plant growth-promoting bacteria (PGPB) (Wang et al., 2021). The primary intent of this study was to identify and maximize siderophores produced by the *P. monteilii* strain MN75947 recovered from a *D. sissoo* plantation forest and investigate its biocontrol ability against fungus species causing mortality in the same tree system. In the first-stage optimization experiment, the effects of pH, Fe concentration, carbon source, nitrogen source, and heavy metals on the siderophore production ability of strains were probed. For the parameters selected from the first stage, the second-stage optimization was performed using a Box–Behnken design in response surface methodology. This potential method allowed us to study the effect of multivariable parameters at a time and their interaction on siderophore production (Baş and Boyaci, 2007). This statistical approach also allowed us to evaluate effective factors responsible for siderophore production in a *P. monteilii* strain (MN75947) and generate optimum conditions for the exclusive factors for the desired response (Abo-Zaid et al., 2020). Partial purification of the culture supernatant by XAD-2 column chromatography was performed, and further siderophore-like compounds were identified *via* LC-MS and FTIR. Since the application of siderophore-producing bacteria has tremendous potential in the maintenance of sustainable agriculture, it could be an eco-friendly substitute for hazardous chemical pesticides. Moreover, siderophores have been reported to play an important role in immobilizing the metal from the metal-contaminated soil, which is toxic for most plants (Trindade et al., 2021; Hardcore et al., 2022). Also, siderophore-assisted bioremediation of toxic contaminants holds promise (Rani et al., 2022). Commercialization of siderophore-producing bacteria as bio-inoculants will thus enable the control of several fungal plant diseases by depriving iron-scavenging pathogens (Basu et al., 2021; Malgioglio et al., 2022) and holds the promise of achieving sustainable agriculture goals.

## Material and methods

### Bacterial strain used in the study

A *Pseudomonas monteilii* B8 strain **(**GenBank accession number MN759447) previously isolated from the *D. sissoo* forest ecosystem, Agroforestry Research Centre, G.B. Pant University of Agriculture and Technology, Pantnagar (28°58′N 79°25′E/28.97°N 79.41°E), was used in this study.

### Quantitative assay for siderophore production

The strain was grown in Luria-Bertani (LB) broth at 30°C and 120 rpm for 48–72 h and spectrophotometrically screened for siderophore production. Production of siderophores was confirmed by the CAS agar test, developed by Schwyn and Neilands (1987) and modified by Alexander and Zuberer (1991). The amount of siderophores was calculated and represented as % siderophore units using the formula % Siderophore = Ar – As/Ar^*^100 (Kumar et al., 2017), where Ar is the absorbance of the reference (CAS reagent) and As is the absorbance of the sample at 630 nm. Siderophore production was confirmed by the qualitative CAS agar test. The log-phase bacterial culture was spotted on nutrient agar plates amended with the CAS solution. The plates were incubated at 28°C under dark conditions for 3–5 days. The appearance of yellow to orange zones confirms siderophore production. All the assays were carried out in triplicates.

### Optimization of conditions for siderophore production *via* a single-factor experiment

Varying sources of carbon and nitrogen, pH, and concentration of iron and heavy metals were optimized for enhanced siderophore production.

### Effect of pH

The effect of varying pH (3–11, with an interval of 2) on siderophore production was studied. The succinate broth at different pHs was inoculated with the log-phase bacterial culture separately and incubated at 37°C for 48–72 h at 120 rpm. Thereafter, 1 ml of culture filtrate was added to 1 ml of the CAS solution; absorbance was measured at 630 nm; and % siderophore units were calculated (Soares, 2022).

### Effect of iron concentration

The succinic acid medium was supplemented with varying concentrations of iron to determine the threshold level of iron that repressed siderophore production. The log-phase bacterial culture was inoculated separately in the succinate broth amended with varying FeCl_3_·6H_2_O concentrations (0, 25, 50, 100, and 150 μM) and incubated at 37°C for 48–72 h at 120 rpm. Thereafter, 1 ml of the culture filtrate was added to the CAS solution (1 ml), and absorbance was measured at 630 nm. The siderophore yield was calculated as % siderophore units (Patel, 2022).

### Effect of carbon and nitrogen sources on siderophore production

The succinate broth (100 ml) was supplemented with 1 g/l of four different carbon sources. The bacterial isolate was inoculated separately in the succinate broth supplemented with sucrose, glucose, starch, and mannitol and incubated at 37°C for 48–72 h at 120 rpm. After 72 h, 1 ml of the culture filtrate was added to 1 ml of the CAS solution (1:1). The absorbance was measured at 630 nm, and %siderophore units were calculated (Waday et al., 2022).

Similarly, a loopful of the log-phase bacterial culture was inoculated into the succinate broth supplemented with ammonium nitrate, yeast extract, protease peptone, and potassium nitrate separately. The flasks were incubated at 37°C for 48–72 h at 120 rpm, after which 1 ml of the culture filtrate was added to 1 ml of the CAS solution; the absorbance was measured at 630 nm; and %siderophore units were calculated (Cornu et al., 2022).

### Effect of heavy metals on siderophore production

To evaluate the influence of heavy metals on siderophore production, the succinate broth (100 ml) was supplemented with 10 μm of HgCl_2_, MnCl_2_, CdCl_2_, and NiCl_2_, separately, followed by incubation at 37°C for 48–72 h. Then, % siderophore units were estimated (Sayyed et al., 2010; Patel et al., 2018; Wang et al., 2022).

### Statistical analysis

Data were analyzed using a two-way ANOVA under a completely randomized design. Each of the parameters tested significantly affects siderophore production. Moreover, the interaction of each parameter with the isolates is also highly significant (Hesse et al., 2018).

### Optimization of siderophore production conditions using response surface methodology

In total, three significant factors influencing siderophore production, namely, NH_4_NO_3_, Fe concentration, and pH 7, were analyzed using the Box–Behnken design of SPSS 19.0 software (SPSS Inc., USA). The appropriateness of the model was checked by using the coefficient of determination (R^2^), lack of fit, and Fisher's F-test, where growth (Y1) and siderophore production (Y2) were considered dependent variables and pH (X_1_), iron concentration (X_2_), and ammonium nitrate (X_3_) were independent variables (RajeNimbalkar et al., 2022).

The following Box–Behnken design equation was used to analyze the correlation between the dependent and independent factors.


(1) $$\begin{array}{r} Y=\beta_{\text{O}}+\sum_{\text{i}=1}^{\text{n}}\beta iXi+\sum_{\text{i}=1}^{\text{n}-1}\overset{\text{n}}{\underset{\text{j}=\text{i}+1}{\sum }}\beta_{\text{ij}}X_{\text{i}}X_{\text{j}}\\ +\sum_{\text{i}=1}^{\text{n}}\beta_{\text{ii}}X^{2}i \end{array}$$

where Y is the predicted response (dependent variable) influenced by independent variables Xi and Xj, and βo, βi, βii, and βij are the constant, linear, quadratic, and cross-product regression coefficients of the models, respectively (Zaghloul and Hamed, 2022).

### Siderophore profiling of *P. monteilii* B8

#### Batch culture of *P. monteilii* B8

To obtain enough purified siderophores for chemical characterization, a large volume of culture was grown in the optimal medium. Typically, 1–2 L of the medium was prepared, and each liter was inoculated with 10 ml of the seed inoculum. The bacterial strain was grown for 24 h at 28°C in a rotatory shaker. After incubation, the culture supernatant was acidified to pH 2.0 with 6M HCl to make the siderophore less soluble in water (Abo-Zaid et al., 2020).

#### XAD-2 column chromatography

Dry resin was taken in a 500-ml beaker, a sufficient quantity of methanol was added to it to cover the resin bed, and the beaker was allowed to stand for 15 min. Methanol was decanted with the simultaneous pouring of water, and again the beaker was allowed to stand for 15 min, after which it was constantly stirred to prevent resin dehydration and bed channeling. Resin slurry was added to the column filled up to 2.5 cm with deionized water so that the column is half filled, and excess water is decanted to allow room for resin swelling. Once the column was prepared, bed volume was calculated by using the following formula (Sayyed and Chincholkar, 2006; Sayyed et al., 2011; Awasthi and Datta, 2019):

The volume of the cylinder = Ω × (1/2 inside diameter)2 × the length of the bed

#### Purification

The harvested cell-free supernatant was subjected to siderophore purification using ethyl acetate. Ethyl acetate extract of the culture supernatant was subjected to XAD-2 column chromatography. The acidified supernatant was passed through the column, and the flow-through was collected. After the entire run of the supernatant, the column was washed with two bed volumes of double-distilled water, and the wash was collected in a separate beaker. Approximately 250 ml of 50% (v/v) methanol was eluted through the column, and 12 fractions of 20 ml of the eluted solution were collected. The flow-through was collected until it turned colorless. To re-equilibrate, the four-bed volumes of methanol, followed by four-bed volumes of double-distilled water, were run through the column. All the fractions obtained were tested by using a CAS reagent; the fractions which appeared yellow to brown were pooled together and concentrated in a rotary evaporator set at 50°C. The dried sample was re-dissolved in 2 ml of distilled water and stored at 4°C.

#### FT-IR and nuclear magnetic resonance analysis

The pure siderophores were analyzed by FT-IR spectrophotometry (FT-IR-8400, Shimadzu). The powder of pure siderophores was dissolved in double-distilled water and analyzed by nuclear magnetic resonance (NMR) (Varian Mercury 300 MHz). The chemical shift was given in δ values (ppm) relative to HOD, and the coupling constants were expressed as *J* values in hertz. Standard programs were used for ^1^H^−1^H chemical shift correlation spectroscopy (COSY), and the molecular structure was determined by LC-MS (Handore et al., 2022).

### Determination of siderophore-mediated *in vitro* inhibition of four fungi strains isolated from the *D. sissoo* forest ecosystem

A total of four shisham plant pathogens, namely, *A. calidoustus, F. oxysporum, T. pinophilus*, and *T. verruculosus*, were screened on half-strength potato dextrose agar. An agar plug (1 × 1 cm) taken from each actively growing *A. calidoustus, F. oxysporum, T. pinophilus*, and *T. verruculosus* strain was placed at the center of the prepared PDA Petri plates. Simultaneously, *P. monteilii* strain was streaked 2 cm away from the agar plug toward the edge of the Petri plates. The plate inoculated with fungus alone served as a control plate. The plate was incubated at 30°C until fungal mycelia completely covered the agar surface of the plate. The same experiment was conducted using the methanol extract of siderophores isolated *via* column chromatography in order to check the specificity of siderophore-mediated antagonism. The percent mycelium inhibition was calculated as follows:


$$\begin{array}{l} Percent\;inhibition\;of\;mycelia=\left(dc-dt/dc\right)\times \;100, \end{array}$$

where dc is the average diameter of the fungal colony in the control group and dt is the average diameter of the fungal colony in the treatment group **(**Karličić et al., 2021).

## Results

### Identification of the potential siderophore-producing isolate

The potential siderophore-positive bacterial isolate was identified with 16S rDNA sequencing analysis. The isolate B8 was found to be 93.13% similar to *P. monteilii*. Upon quantitative and qualitative estimations, the strain B8 (MN759447) was found to produce siderophores in the range of 80.36 ± 0.005 siderophore units.

### Effect of growth parameters on siderophore production in *P. monteilii* B8

**Figure 1:**
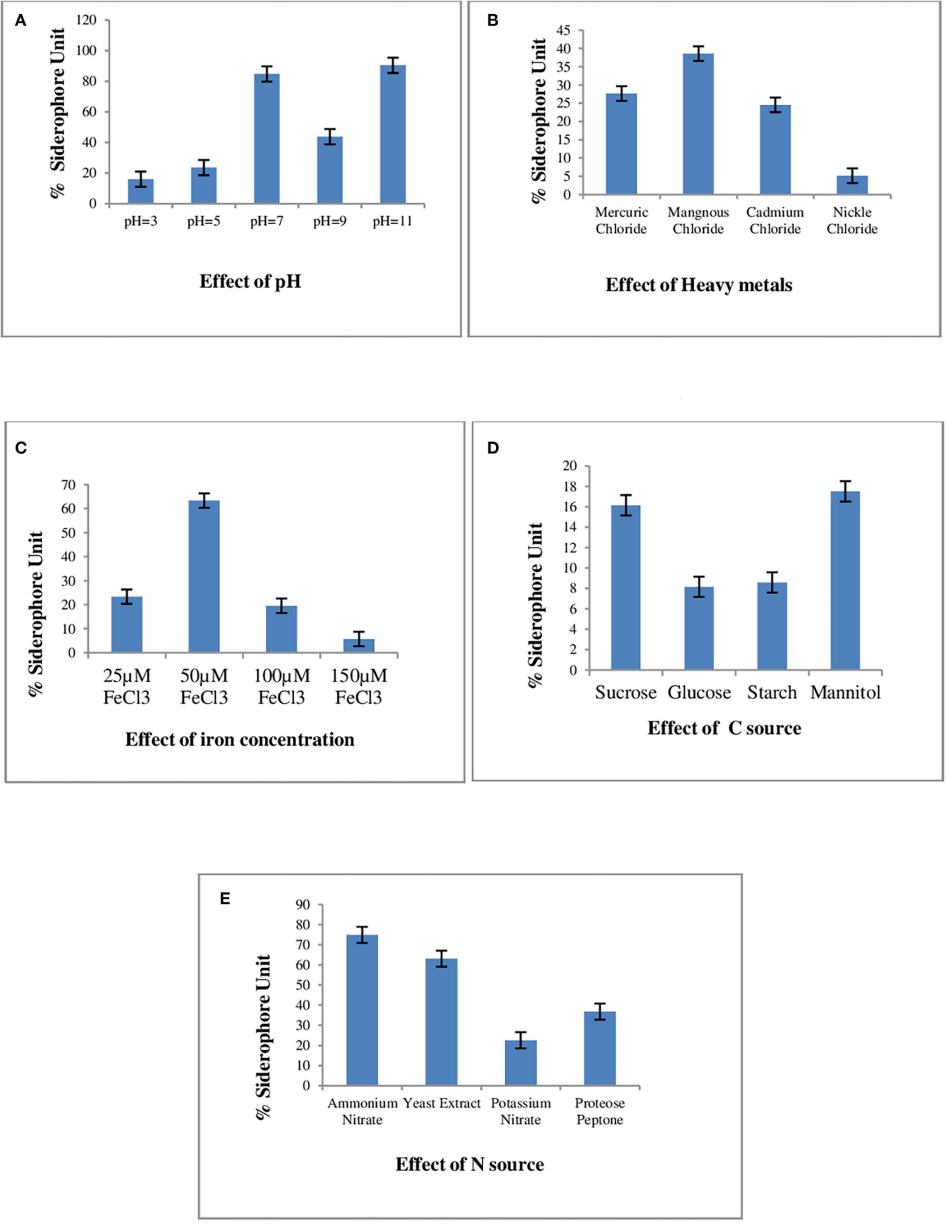
Effect of various culture conditions on Siderophore Production in *P. monteilli* strain B8 (MN759447). The succinate broth was inoculated with log phase of strain *P. monteilli* strain B8 (MN759447) and incubated at 37°C for 48–72 h at 120 rpmand amended separately with different heavy metals, carbon source, nitrogen source, Fe concentration and at different pHs. The %SU was calculated using formula = Ar – As/Ar × 100 [where, Ar = Absorbance of the reference (CAS Reagent); As = absorbance of the sample at 630 nm]. All the assays were carried out in triplicates, analyzed using two ways ANNOVA under completely randomized design. **(A)** Effect of pH, **(B)** effect of heavy metals, **(C)** effect of iron concentration, **(D)** effect of C source, **(E)** effect of N source.

The varying sources of carbon and nitrogen, pH, and concentration of iron and heavy metals for enhanced siderophore production in the *P. monteilii* strain MN759447 were optimized. The production of siderophores in the *P. monteilii* strain MN759447 varied from 16.01 to 84.85% SU at pH 3–11. The trend line in Figure 1A indicates that pH 7 is optimum for siderophore production in *P. monteilii* with a maximum recovery of 84.85% SU. Of the four nitrogen sources tested, only ammonium nitrate and yeast extract supported siderophore production, yielding up to 74.91 and 63.11% SU, respectively, as shown in Figure 1E. Siderophore biosynthesis in *P. monteilii* was not supported by any of the four carbon sources tested (sucrose, glucose, starch, and mannitol),as the yield was less than 20% SU in all four carbon sources, as given in Figure 1D. Figure 1C depicts that an iron concentration of 50 μM is optimal for siderophore production. The succinate broth amended with MgCl_2_, CdCl_2_, NiCl_2_, and MnCl_2_ yielded 27.65, 24.56, 5.16, and 38.38% SU, respectively, as shown in Figure 1B. The siderophore yield was highest in the succinate broth amended with MnCl_2_.

### Selection of siderophore-producing conditions using the single-factor experiment

Growth conditions, including an optimal pH of 7–7.5, an Fe concentration of 50 μm, and the addition of NH_4_NO_3_, were most suitable for siderophore production in *P. monteilii* MN759447, obtaining values up to 85 % SU. The second-stage optimization employs a Box–Behnken design in RSM (Design Expert 10 software, State Ease In., USA).

### Regression analysis of bacterial strain B8 on growth

The values of R^2^ and adj R^2^ for isolate B8 were 0.9688 and 0.92, respectively. These values are reasonably close to 1. Moreover, the R^2^ value (0.9688) implies that the model accounted for 96.88% of the data and that the residual error is less, indicating a high correlation between the observed and the predicted value (a table for regression analysis is provided in the Supplementary Table 1). The following equation is a second-order polynomial equation describing the effect of significant variables on bacterial growth:

OD at 600 nm of isolate B8 = +0.34+0.13X_1_ + 0.25X_2_ + 0.19X_3_ – 0.24X_1_X_2_ – 0.33X_1_X_3_ + 0.25X_2_X_3_ – 0.067${\text{X}}_{1}^{2}$ + 0.28${\text{X}}_{2}^{2}$ – 0.15${\text{X}}_{3}^{2}$

The sign and magnitude of the coefficients in the previous equation indicate the effect of the significant independent variables on growth. The effects of pH (X_1_), iron concentration (X_2_), and ammonium nitrate concentration (X_3_) on growth were highly significant (*p* < 0.01). This signifies that the OD at 600 nm increased with an increase in pH, ammonium nitrate concentration, and iron concentration. At the interactive level, the interaction between pH and iron concentration (X_1_X_2_), iron concentration and ammonium nitrate concentration (X_2_X_3_), and pH and ammonium nitrate (X_1_X_3_) had a significant positive effect on siderophore production. At the quadratic level, the concentration of Fe (X_3_) had a significant negative (*p* < 0.01) effect on bacterial growth, which is indicated by a decrease in absorbance at 600 nm.

### Growth of *P. monteilii* B8 as influenced by independent variables

#### Effect of pH

**Figure 2:**
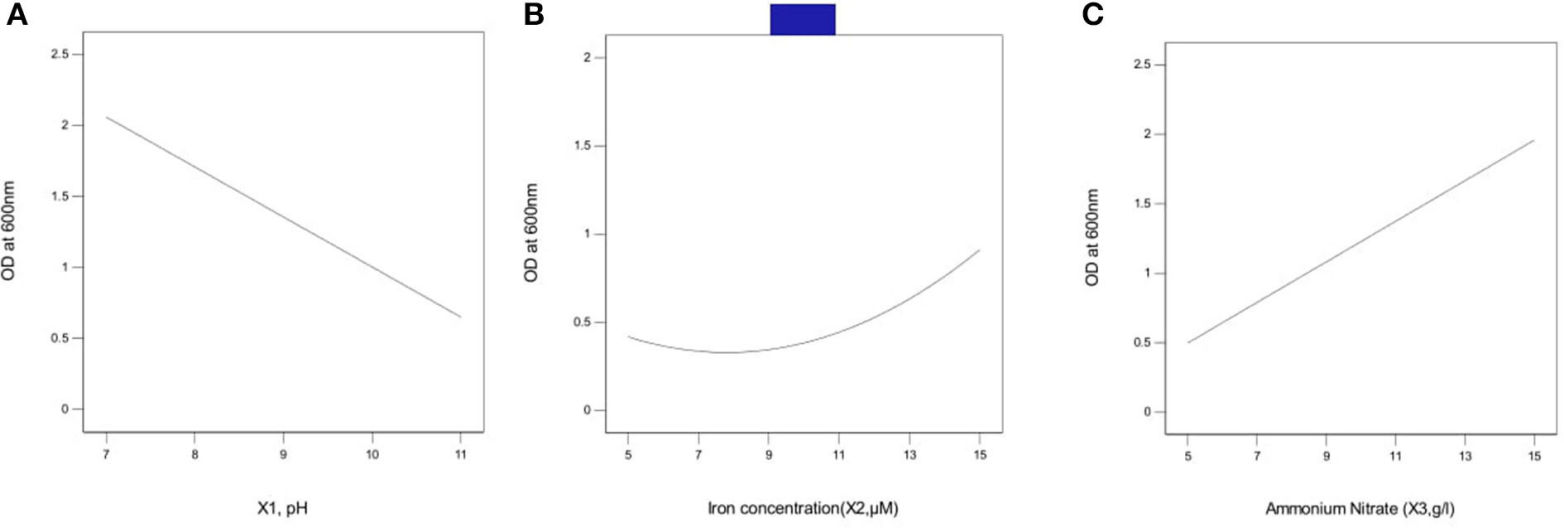
Effect of independent variables on growth in bacterial isolate B8 **(A)** pH, **(B)** iron concentration, and **(C)** ammonium nitrate concentration. Initially at pH 7 the OD was the densest as the pH raises above 7 a steep decline in growth was observed. At iron concentration between 5 and 9 μM, a linear growth was observed. As the concentration rises above 9 μM, OD starts increasing and increased up to iron concentration of 15 μM. An exponential rise in OD was observed as the ammonium nitrate concentration was increased above 5 g/l. The assays were carried out in triplicates, analyzed using Box Behnken Design (response surface methodology).

The effect of pH on growth was tested at optimal values of Fe concentration (15 μm, X_2_) and ammonium nitrate concentration (15 g/l, X_3_). With an increase in pH, absorption (OD) values decreased, as shown in Figure 2A. Initially, the OD was the densest at pH 7. As the pH increased above 7, a steep decline in growth was observed.

#### Effect of Fe concentration

A linear growth was observed at an iron concentration between 5 and 9 μM. OD started increasing as the iron concentration increased between 9 and 15 μM. Therefore, iron concentrations of 5–15μM supported the growth of isolate B8 (Figure 2B).

#### Effect of NH_4_NO_3_ concentration

The effect of ammonium nitrate concentration was tested at optimal value of (pH 7, X_1_) and Fe concentration (15 μm, X_2_) (Figure 2C). An exponential rise in OD was observed above 5g/l NH_4_NO_3_. Initially, the OD was the densest at pH 7. As the pH increased above 7, a steep decline in growth was observed.

#### Regression analysis of siderophore production in strain B8

The value of the R^2^ for siderophore production on isolate B8 was 0.9988, and the Adj R^2^ value, which was found to be 0.98, was reasonably close to 1, which implies that the model could account for 99.88% of the data with less residual error (a table for regression analysis is provided in the Supplementary Table 2). The following equation is a second-order polynomial equation describing the effect of significant variables on siderophore production:

% Siderophore unit in isolate B8 = +68.72 – 10.02X_1_ – 9.20X_2_ + 12.11X_3_ – 8.09X_1_X_2_ −11.01X_1_X_3_ + 5X_2_X_3_ – 26.83${\text{X}}_{1}^{2}$ + 4.04${\text{X}}_{2}^{2}$ – 25.25${\text{X}}_{3}^{2}$

All three variables in the previous equation had a highly significant (*p* = 0.01) positive effect on siderophore production. This signifies that the % siderophore unit increases with an increase in pH, ammonium nitrate concentration, and iron concentration. At the interactive level, the interaction between pH and iron concentration (X_1_X_2_), iron concentration and ammonium nitrate concentration (X_2_X_3_), and pH and ammonium nitrate concentration (X_1_X_3_) had a significant negative effect on siderophore production, whereas, at the quadratic level, the concentration of Fe (X_2_), pH (X_1_), and ammonium nitrate (X_3_) had a significant positive (*p* < 0.01) effect on siderophore production.

### Siderophore production in *P. monteilii* B8 as influenced by independent variables

#### Effect of pH

**Figure 3:**
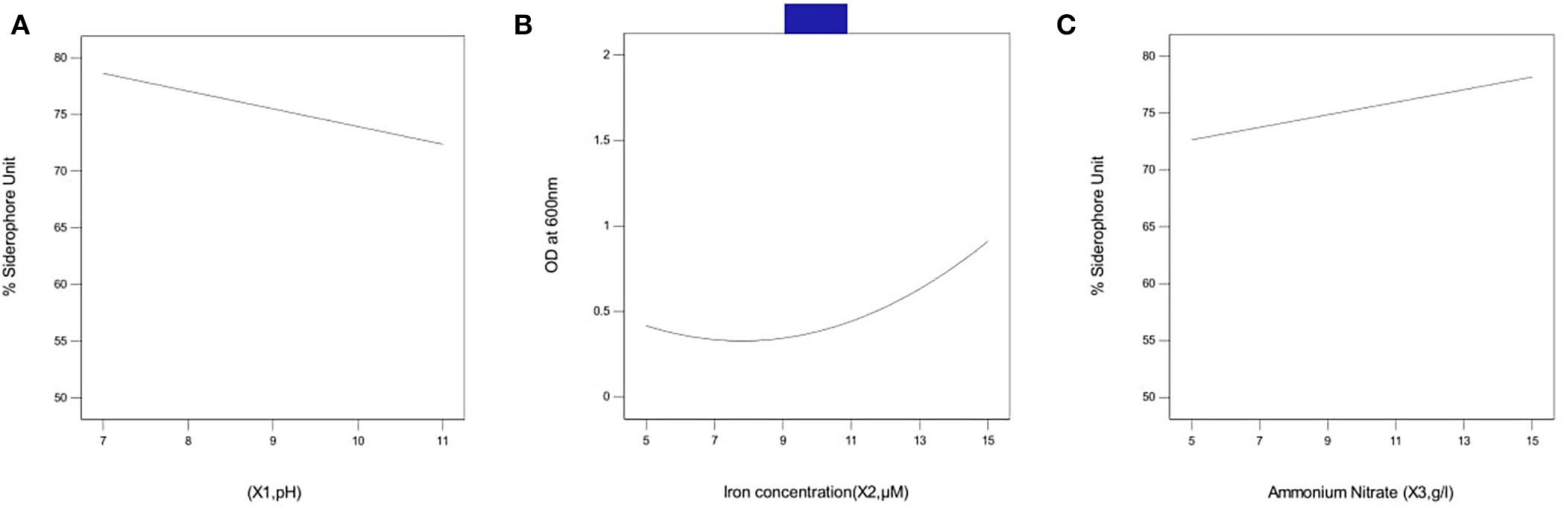
Effect of independent variables on siderophore production in bacterial isolate B8 **(A)** pH, **(B)** iron concentration, and **(C)** ammonium nitrate concentration. pH 7 was found optimum for siderophore production (80%SU) whereas upon increasing the pH, siderophore production starts declining attaining a value of 74% at pH 11. A value above 80% was obtained at 15 μM Fe concentration %SU increased from 73 to 85% when the concentration of NH_4_NO_3_ was increased above 10 g/l. The assays were carried out in triplicates, analyzed using Box Behnken Design (response surface methodology).

Figure 3A represents the linear effect of pH on siderophore production in *P. monteilii* B8. At pH 7, optimum siderophore production was found (80% SU), but, with the increase in pH, siderophore production started declining, attaining a value of 74% at pH 11. The decline in siderophore production at alkaline pH explains the solubilization and bioavailability of iron in the medium.

#### Effect of Fe concentration

Figure 3B represents the linear effect of iron concentration on siderophore production in *P. monteilii* B8. A value above 80% was obtained at an Fe concentration of 15 μM. Siderophore production substantially increased with an increase in the Fe concentration above 5 μM.

#### Effect of NH_4_NO_3_ concentration

Figure 3C represents the linear effect of NH_4_NO_3_ concentration on siderophore production in *P. monteilii* B8. The % SU increased from 73 to 85% when the concentration of NH_4_NO_3_ increased above 10 g/l.

#### Interactive effect of independent variables on siderophore production in *P. monteilii* B8

**Figure 4:**
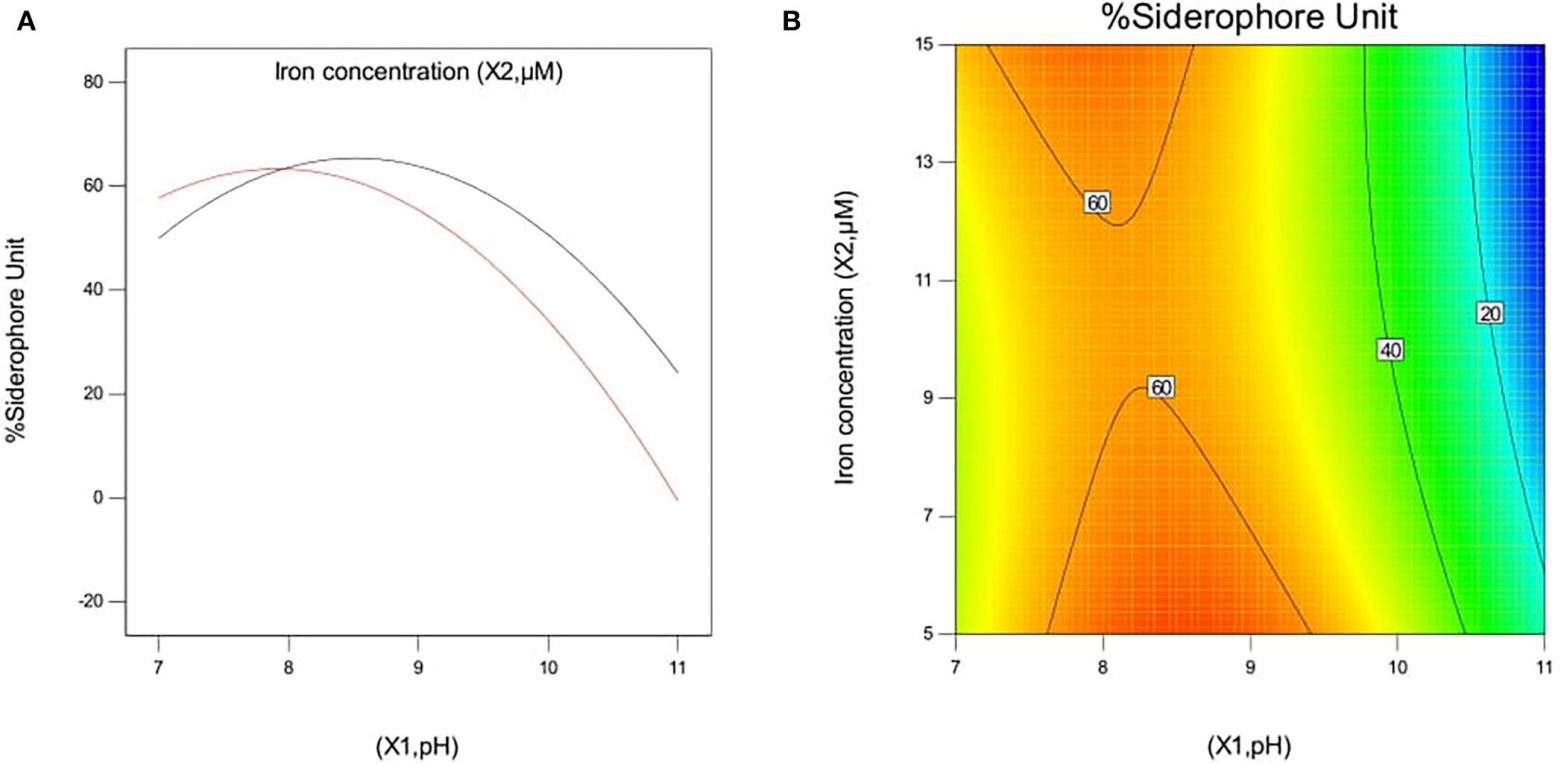
Effect of pH and iron concentration on siderophore production In isolate B8 at optimized condition of ammonium nitrate concentration (X3, 15 g/l). **(A)** Interactive and **(B)** contour.

**Figure 5:**
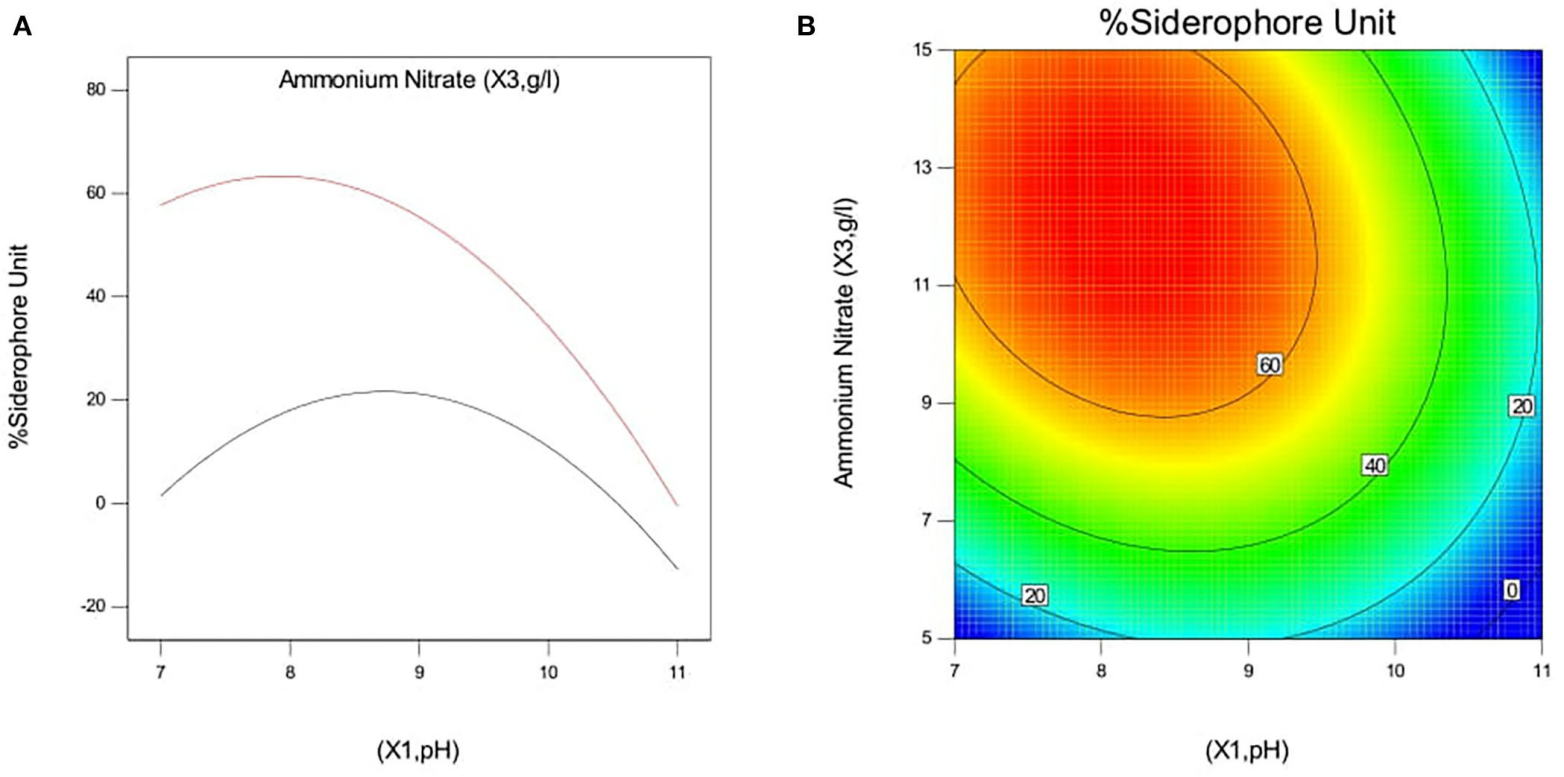
Effect of pH and ammonium nitrate on siderophore production In isolate B8 at optimized condition of iron concentration (X2, 15 μM). **(A)** Interactive and **(B)** contour.

**Figure 6:**
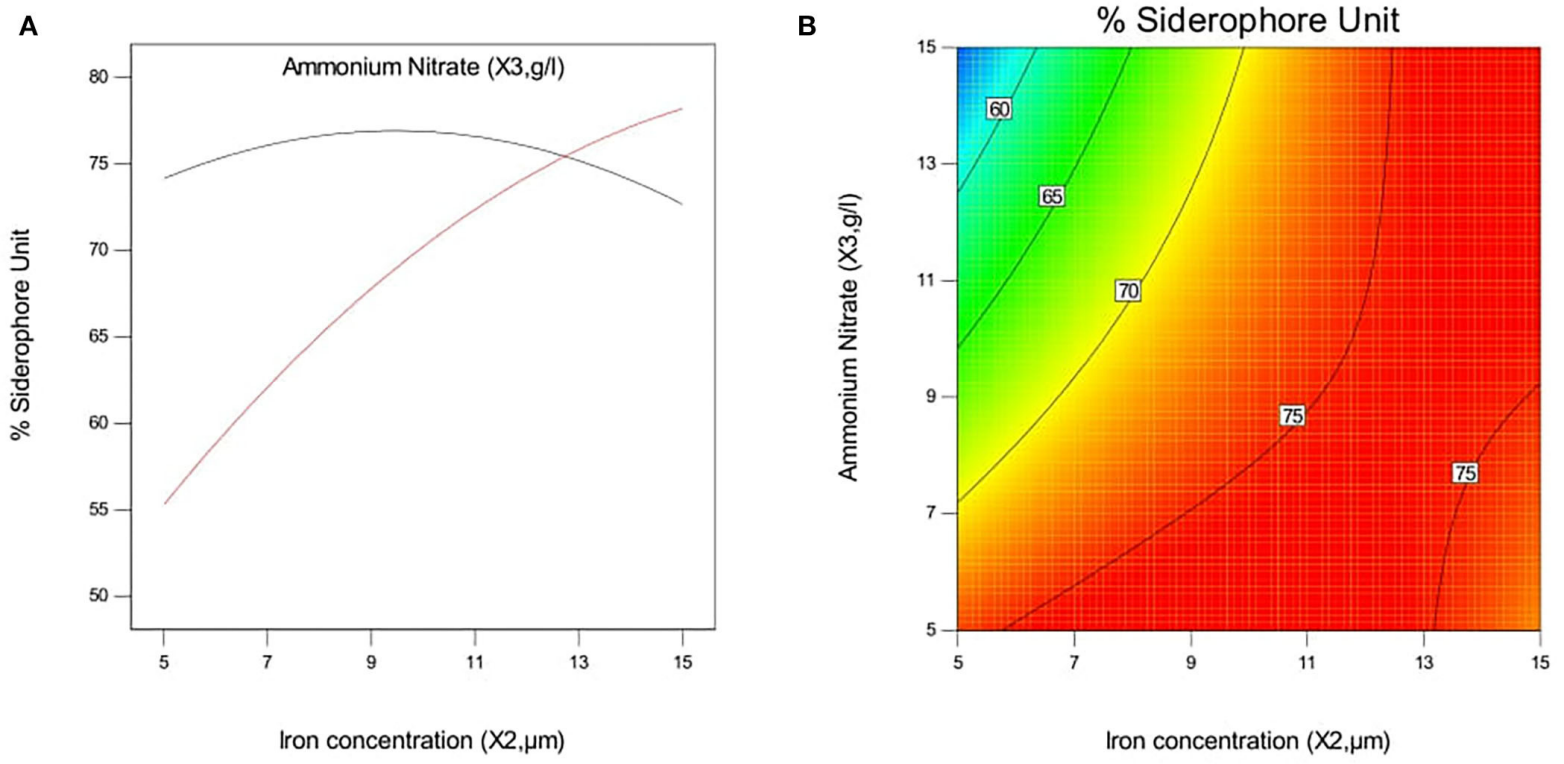
Effect of iron concentration and ammonium Nitrate concentration on siderophore production in *P. monteilli* B8 at optimized condition of pH (X1, 7.29). **(A)** Interactive and **(B)** contour.

**Figure 7:**
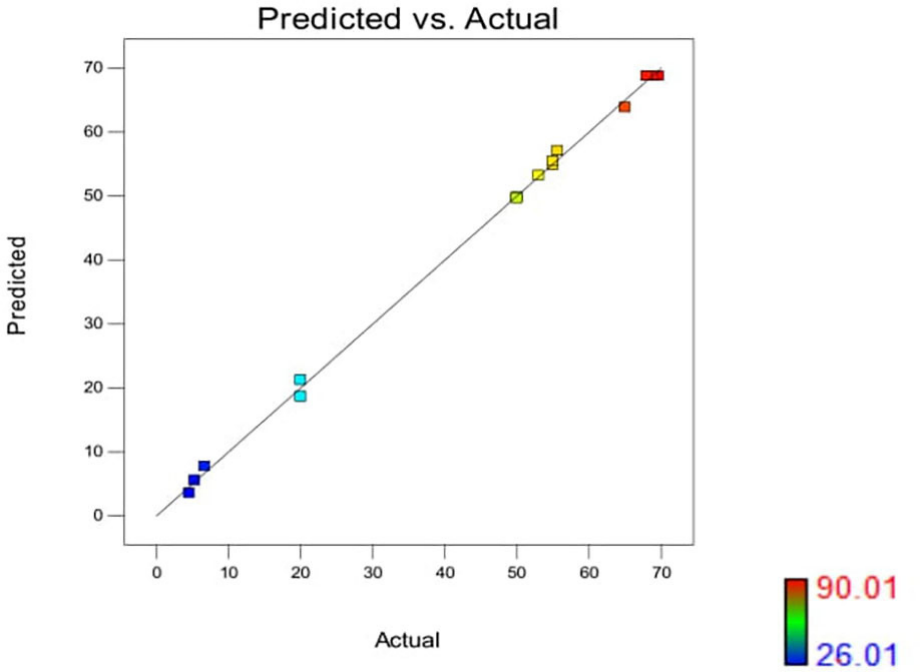
Color points by value of %SU in *Pseudomonas monteillii* (actual vs. predicted). Upon experimentation, actual values for maximum bacterial growth were falling in line with the predicted values, i.e., pH = below 9, Fe = 9–15 μM, NH_4_NO_3_ 15 g/l, whereas for siderophore production, actual values were also somehow similar to predicted values,·pH = below 7, Fe = 7–13 μM , NH_4_NO_3_ 15 g/1, and 89.9% siderophore units was recovered.

The effect of pH and iron concentration on siderophore production in isolate B8 at an optimal ammonium nitrate concentration (X_3_, 15 g/l) is shown in Figures 4A,B. Figures 5A,B show the effect of pH and ammonium nitrate on siderophore production in isolate B8 at an optimal iron concentration (X_2_, 15M), with (a) and (b) showing interactive and contour effects. Figures 6A,B show the effect of ammonium nitrate concentration on siderophore production at optimal pH (7.29) and iron concentration (15 g/l). The % siderophore unit increases with the increase in the ammonium nitrate concentration above 7 g/l, reaching a value above 89% SU. Upon experimentation, for maximum bacterial growth, actual values were in line with the predicted values, with a pH value below 9, an Fe concentration of 9–15 μM, and an NH_4_NO_3_ concentration of 15 g/l, whereas for siderophore production, actual values were somehow similar to predicted values, with a pH value below 7, an Fe concentration of 7–13 μM, and an NH_4_NO_3_ concentration of 15 g/l, and 89.9% of siderophore units were recovered, as shown in Figure 7.

#### FT-IR spectrum of *P. monteilii* B8

The functional groups present in the FTIR spectrum of *P. monteilii* were N–H (amines), –OH alcohol, C–O stretch, and C=O stretch (infrared spectrum is provided in the Supplementary Figure 1). Peaks were obtained at 3,189, 2,360, 1,602, and 1,566 wave numbers, which indicates the presence of one C–H bending with the functional group –CH_2_ and one –N–O structure due to functional group N–O bonding, which was present in pseudomonine siderophores, which are a low-affinity Fe^3+^-binding siderophores.

#### LC-MS of methanol extract for untargeted siderophore profiling

From LC-MS chromatography for *P. monteilii*, all 14 compounds detected (Table 1) (chromatogram of the compounds identified is given in the Supplementary Figure 1) showed a compound having a molecular weight similar to that of pseudomonine, that is, 329.183 g/mol, with a retention time of 0.63 min. This could be a derivative of pseudomonine 2(S)-2[1 tert-butoxycarbonyl amino]-5-(cyclohexyloxy)-5 oxopentanoic acid (C_16_H_27_NO_6_). The second derivative identified in the methanol extract was 2,2,6,6 tetramethyl 4-piperidinyl heptanoate (C_16_H_31_NO_2_), with a molecular weight of 269.234 g/mol and a retention time of 8.07 min. Several other compounds, like 1 methyl 1H pyrrole carbonate (C_6_H_6_N_2_), salicylic acid (C_6_H_9_N_3_), and kynurenic acid, were also reported in the methanol fraction of purified siderophores (Table 1). The *Pseudomonas* siderophore quinolobactin is synthesized from xanthurenic acid, an intermediate of the kynurenine pathway (Giannelli et al., 2022). The detection of kynurenic acid could be an intimation of the presence of quinolobactin siderophores in the methanol extract, but this could not be detected due to a lower retention time in LC-MS profiling. Moreover, bacterial salicylate production is associated with the biosynthesis of small ferric ion-chelating molecules, salicylic acid-derived siderophores (known as catecholates), under iron-limited conditions (Mishra and Baek, 2021). To confirm siderophore-mediated definite antagonism by *P. monteilii*, the methanol extract was also used to check the antagonistic efficacy against the four fungal strains, and the results were similar to those of *in vitro* dual-culture plate assay.

**Table 1:** Compounds detected in LC-MS in strain *Pseudomonas monteillii*.

**S. no**.	**Compound name**	**Chemical formula**	**Molecular weight**	**Annotation source (mzclound) best % similarity**	**Retention time**	**Retention area**
1	2,2,6,6 Tetremethyl 4-piperdinyl heptanoate	C_16_H_31_NO_2_	269.234	80	8.07	16,043.35
2	2(S)-2[1 tert-Butoxycarbonyl amino]-5-(cyclohexyloxy)-5 oxopentanoic acid	C_16_H_27_NO_6_ *a pseudomonine derivative	329.183319	98.9	0.63	12,519.37
3	1 methyl 1H-Pyrrole 2 carbonate	C_6_H_6_N_2_	106.05306	97.9	98.17	7,305,307.02
4	2-amino 4,6 dimethyl pyrimidine	C_6_H_9_N_3_	123.07961	98.47	98.47	36,178,261
5	Salicyclic acid	C_7_H_6_O_3_	138.031	98.4	2.91	993,547.04
6	Pthalic anhydride	C_8_H_4_O_3_	148.015	96	3.08	6,038,923.21
7	Benzoxazole	C_7_H_5_NO	119.037	90.5	0.93	406,152.63
8	Dipropyl phthalate	C_9_H_21_N_5_O_5_	279.1545	93.2	4.00	401,448.52
9	Pyrazinamide	C_5_H_5_N_3_O	123.04308	97.1	99.07	352,609.36
10	Kynurenic acid	C_10_H_7_NO_3_	189.04222	83	0.98	966,778.94
**11**	Isonicotinamide	C_6_H_7_N_3_	121.06377	77.9	98.47	906,505.89
12	1 Amino 2,6 dimethyl pyridinium	C_7_H_11_N_2_	123.09193	97.2	99.48	18,954.83
						
13	Butyrophenone	C_10_H_12_O	148.08861	87.1	2.45	242,801.80
14	Methyl histidine	C_7_H_11_N_2_	123.09193	97.2	99.48	189,543.83

#### Antagonistic activity of *P. monteilii* B8

**Figure 8:**
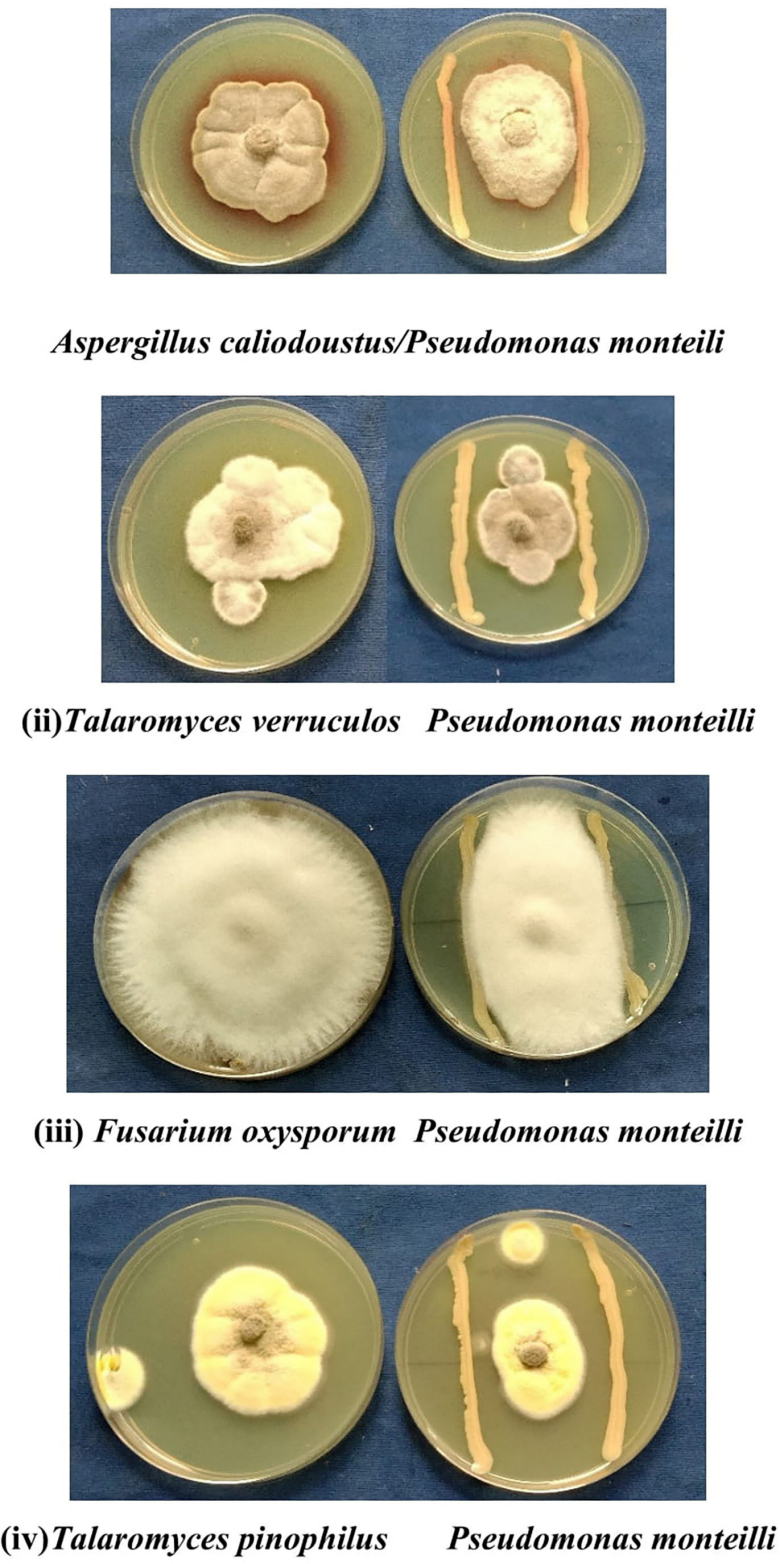
Four representative examples of *in vitro* dual culture assays against fungus (i) *Aspergillus calidoustus*, (ii) *Talaromyces verruculosus*, (iii) *Fusarium oxysporum*, (iv) *Talaromyces pinophilus* for bacterial isolate *Pseudomonas monteilli*. It varied from 41 to 65% against different fungus such as *A. calidoustus* (65%), *F. oxysporum* (41.66%), *T. pinophilus* (65%), *T. verruculosus* (65.1%).

Broad-spectrum antagonistic activity of *P. monteilii* bacteria was observed against the four fungal strains isolated from the *Dalbergia sissoo* Roxb. forest ecosystem in the Terai region of the Himalayas. It varied from 41 to 65% against the four fungi, namely, *A. calidoustus* (65%), *F. oxysporum* (41.66%), *T. pinophilus* (65%), and *T. verruculosus* (65.1%) (Figure 8).

## Discussion

Iron liberation in the soil in a bioavailable form that can be easily taken up by plants is dependent on redox potential and soil pH. In the soil, an oxidative environment with a high pH predominates, allowing the synthesis of Fe oxides and thereby lowering its bioavailability. Sulochana et al. (2014) found that *Pseudomonas* strain JAS-25 produces the highest number of siderophores at pH 7 (130 μM) and that a single unit increase in pH lowers siderophore production by 5%. The influence of pH on siderophore production in strains SID 30 and SX9 was recently examined by Wang et al. (2021) and Reddy Kiran Kalyan et al. (2022), and pH values of 7–8 were determined to be optimal for siderophore formation. Many studies showed that ammonium sulfate and nitrate salts are potential sources of nitrogen for siderophore biosynthesis. The siderophore production efficiency in strains VITVK5 and VITVK6 was higher than 60% SU when sodium nitrate was used as a nitrogen source. Furthermore, nitrogen source optimization reveals that both organic and inorganic nitrogen sources [(NH_4_)_2_ SO_4_, NaNO_3_, and urea] allow siderophore synthesis with a value greater than 60% SU. In addition to nitrogen sources, pH also has a significant impact on iron solubility and reactivity. The spontaneous chemical oxidation of iron can be rapid at neutral pH. However, this abiotic oxidation occurs at a relatively low rate in water at low pH. The concentration of iron III required for optimal bacterial growth is between 10^−6^ and 10^−7^ M (Vraspir and Butler, 2009). Under iron-depleted environments, iron concentration plays a crucial role in the production of siderophores. In the *P. monteilii* strain MN75947, an iron concentration of 50 μM was determined to be ideal for siderophore synthesis. A similar result was obtained where an iron concentration of 30 μM was the minimum requirement for siderophore synthesis in two fluorescent pseudomonads, *P. fluorescens* NCIM 5096 and *P. putida* NCIM 2847 (Sayyed et al., 2005, 2019). According to Tailor and Joshi (2012), a lower iron concentration in the medium would induce higher siderophore production to bind to Fe^3+^ ions and make them available to the cell, but as the iron concentration increases beyond the threshold value, siderophore production falls sharply most likely due to negative transcriptional regulation by FUR proteins, where Fe^2+^ acts as a cation. When the second-stage optimization was performed using the Box–Behnken design of response surface methodology, 17 sets of the experiment were predicted with five central points, where optimal values of pH, NH_4_NO_3_ concentration, and Fe concentration were calculated, which were competent values for enhanced siderophore production and growth in the *P. monteilii* strain (MN759447). To achieve maximum siderophore units, optimal levels of Fe concentration (15M, X_2_), ammonium nitrate concentration (15 g/l, X_3_), and pH (7.29, X_1_) were obtained under their independent and interacting effects (Yu et al., 2017). Also, enhanced siderophore production was reported in *Pseudomonas aeruginosa* RZS9 by applying a two-stage statistical approach, for example, Plackett–Burman design and response surface methodology (RSM), using a central composite design (CCD) (Shaikh et al., 2016a,b). During the experiment, actual values for maximum bacterial growth were in line with the predicted values, with a pH value below 9, an Fe concentration of 9–15 M, and an NH_4_NO_3_ concentration of 15 g/l, whereas for siderophore production, actual values were quite similar to the predicted value of 90.01%, with a pH value below 7, an Fe concentration of 7–13 M, and an NH_4_NO_3_ concentration of 15 g/l; 89.9% siderophore units were achieved (Figure 7), which was 10% higher than the value obtained in CAS quantification (80.06%). After the siderophore production was optimized, the fungal strain was isolated and analyzed to see what sort of siderophore it contained: In *P. monteilii*, a pseudomonine derivative 2(S)-2[1 tert-butoxycarbonyl amino] (5)-(cyclohexyloxy)-5 oxo pentanoic acid (C_16_H_27_NO_6_) having a molecular weight similar to that of pseudomonine, that is, 329.183 g/mol, with a retention time of 0.63 minutes was detected. *Pseudomonas* siderophores that have been identified include pseudobactin, pyoverdine, pyochelin, enantio-pyochelin, pyorubrin, yersiniabactin, achromobactin, quinolobactin, thio-quinolobactin, pyridine-2,6-bis(thiocarboxylic acid) (PDTC), aeruginic acid, and corrugatin **(**Meesungnoen et al., 2021 and Schalk et al., 2020). In *Pseudomonas fluorescens* WCS374r, pseudobactin PSB 374, salicylic acid, and pseudomonine (PSM) were reported as siderophores (Djavaheri et al., 2012). The second pseudomonine derivative reported in this study was identified as 2,2,6,6 tetramethyl-4-piperidinyl heptanoate (C_16_H_31_NO_2_), with a molecular weight of 269.234 g/mol and a retention time of 8.07 min. Other chemicals, such as 1 methyl 1H pyrrole carbonate (C_6_H_6_N_2_), salicylic acid (C_6_H_9_N_3_), and kynurenic acid, were also identified in the methanol fraction of the purified siderophores. The *P. monteilii* B8 strain (MN759447) efficiently inhibited the growth of the four fungi, namely, (i) *A. calidoustus*, (ii) *T. verruculosus*, (iii) *F. oxysporum*, and (iv) *T. pinophilus*, in a dual-culture plate assay. Sayyed and Patel (2011) assayed *in vitro* phytopathogen (*Aspergillus niger* NCIM 1025, *Aspergillus flavus* NCIM 650, *Fusarium oxysporum* NCIM 1281, *Alternaria alternata* ARI 715, *Cercospora arachidicola, Metarhizium anisopliae* NCIM 1311, and *Pseudomonas solanacearum* NCIM 5103) suppression activity of siderophore preparations of Ni- and Mn-resistant *Alcaligenes* sp. STC1 and *Pseudomonas aeruginosa* RZS3 SH-94B. The results revealed that the *P. monteilii* strain MN759447 exhibits a unique plant growth promotion property of producing siderophores, which is one of the key mechanisms in the biological control of plant diseases. Therefore, it can also be developed as a broad-spectrum biofungicide for commercial purposes to limit the high mortality rate caused by pathogenic fungi in *D. sissoo* plantation forests (Shaikh et al., 2016a,b; Hamid et al., 2021; Subba and Mathur, 2022).

## Conclusion

A strain isolated from a *D. sissoo* plantation forest at the Agroforestry Research Centre, Pantnagar, Uttarakhand (28°58′N 79°25′E/28.97°N 79.41°E) possessed the plant growth promotion trait of producing siderophores. A single-factor experiment for the optimization of six parameters, mainly growth media, variable pH range, variable iron concentration, carbon and nitrogen sources, and heavy metals, for enhanced recovery of siderophore production was carried out. After the single-factor experiment, response surface methodology in a statistical software package was used for the optimization of three parameters, namely, pH, iron concentration, and ammonium nitrate concentration, to analyze the effect on dependent variables: growth and siderophore production. Iron concentration, pH, and ammonium nitrate were the most influential factors responsible for increased siderophore production. A pH value below 7, an Fe concentration of 9–15 M, and an NH_4_NO_3_ concentration of 9 g/l were most suitable for the enhanced recovery of siderophore units in *P. monteilii*, whereas a pH value of 7, an Fe concentration below 7M, and an NH_4_NO_3_ concentration below 11 g/l were the optimal growth conditions; 89.9% SU was recovered as predicted in the experiment. After the optimization study, the type of siderophores produced by the strain was identified *via* LC-MS and FTIR. Various iron-scavenging metabolites were identified, of which pseudomonine, salicylic acid, pyrazinamide, and kynurenic acid were reported in the methanol fraction of the siderophore extract. The availability of siderophore-producing bacteria in the rhizosphere is important in agriculture to supply iron to the plants and to prevent the growth of phytopathogens, which are iron-dependent. To our knowledge, this is the first report on siderophore-producing bacteria from the *Dalbergia sissoo* forest ecosystem. The application of siderophore-producing bacteria as bio-inoculant is immensely important for crop fields and tree-based ecosystems to improve yield and maintain the fertility of the soil. The findings of this study are significant.

## Data availability statement

The datasets presented in this study can be found in online repositories. The names of the repository/repositories and accession number(s) can be found below: https://www.ebi.ac.uk/metabolights/, MTBLS5605.

## Author contributions

PS: experimentation, compilation, statistical analysis of data, and preparation of experiments. MS: conceptualization and design of the study. KS: design of RSM for optimization studies and analysis. HE, AG, SA, MA, and RS: formal analysis and writing–review, editing, and revision. All authors contributed to the article and approved the submitted version.

## Acknowledgments

This project was supported by the Researchers Supporting Project (Number RSP-2023R7), King Saud University, Riyadh, Saudi Arabia, and the RMC, Universiti Teknologi Malaysia (UTM), through industrial projects (Nos. R.J.130000.7609.4C465 and R.J.130000.7609.4C359). Research fellowship granted by the Alexander von Humboldt Foundation, Bonn, Germany to AG is also gratefully acknowledged the funding from ICAR-AF to MS and University Ph.D. assistantship to PS is duly acknowledged.

## Conflict of interest

The authors declare that the research was conducted in the absence of any commercial or financial relationships that could be construed as a potential conflict of interest.

## Publisher's note

All claims expressed in this article are solely those of the authors and do not necessarily represent those of their affiliated organizations, or those of the publisher, the editors and the reviewers. Any product that may be evaluated in this article, or claim that may be made by its manufacturer, is not guaranteed or endorsed by the publisher.
