# Supplementary figures and images for: Optimization and identification of siderophores produced by Pseudomonas monteilii strain MN759447 and its antagonism toward fungi associated with mortality in Dalbergia sissoo plantation forests
Source: Front Plant Sci. 2022 Nov 7;13:984522. doi: 10.3389/fpls.2022.984522 (PMC9696734; doi:10.3389/fpls.2022.984522)

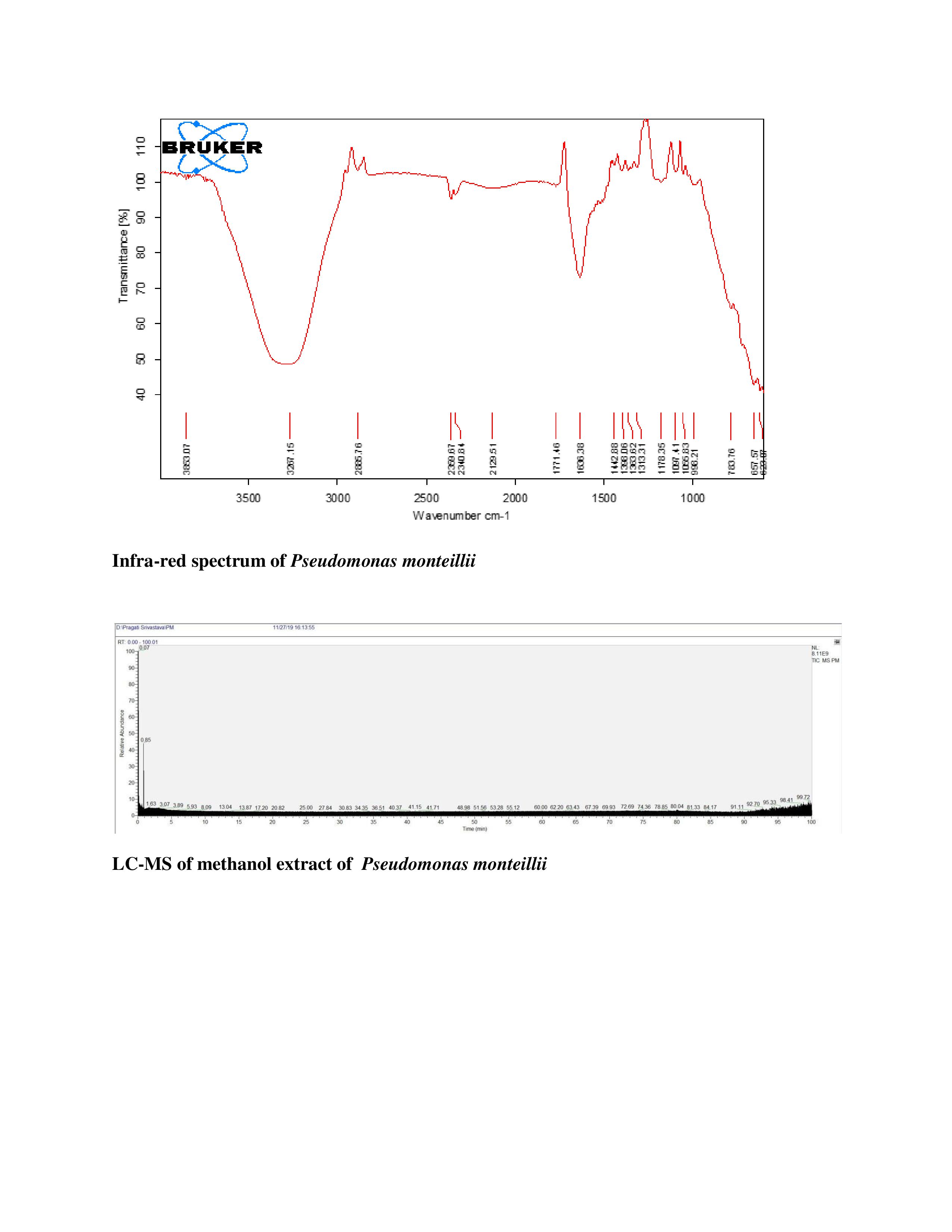

Supplement: Supplementary file 1 [file Image_1.jpeg]
