# Supplementary material for: Optimization and identification of siderophores produced by Pseudomonas monteilii strain MN759447 and its antagonism toward fungi associated with mortality in Dalbergia sissoo plantation forests
Source: Front Plant Sci. 2022 Nov 7;13:984522. doi: 10.3389/fpls.2022.984522 (PMC9696734; doi:10.3389/fpls.2022.984522)
Supplement: Supplementary file 2 [file Table_1.docx]

| **Source** | **Degree of freedom** | **Sum of square** | **Mean of square** | **F-Value (Fischer’s test)** | **p-value (Probability)** |
| --- | --- | --- | --- | --- | --- |
| **Model** | 9 | 9538.79 | 1059.87 | 659.45 | 0.0001 |
| **X_1_-pH** | 1 | 803.40 | 803.40 | 499.88 | 0.0001 |
| **X_2_-Conc of Fe** | 1 | 677.86 | 677.86 | 421.76 | 0.0001 |
| **X_3_-Ammonium Nitrate** | 1 | 1172.97 | 1172.97 | 729.83 | 0.0001 |
| **X_1_X_2_** | 1 | 261.63 | 261.63 | 162.79 | 0.0001 |
| **X_2_X_3_** | 1 | 485.32 | 485.32 | 301.97 | 0.0001 |
| **X_1_X_3_** | 1 | 99.90 | 99.90 | 62.16 | 0.0001 |
| **X_1_^2^** | 1 | 3030.83 | 3030.83 | 1885.78 | 0.0001 |
| **X_2_^2^** | 1 | 68.65 | 68.65 | 42.72 | 0.0003 |
| **X_3_^2^** | 1 | 2684.37 | 2684.37 | 1670.21 | 0.0001 |
| **Residual Error** | 7 | 11.25 | 1.61 |  |  |
| **Lack of Fit** | 3 | 9.44 | 3.15 | 6.96 | 0.0045 |
| **Pure Error** | 4 | 1.81 | 0.45 |  |  |
| **Corrected Total** | 16 | 9550.04 |  |  |  |
| **R^2^** |  | 0.9988 |  |  |  |
| **R^2^adj** |  | 0.9839 |  |  |  |
| **Lack of Fit** |  | significant |  |  |  |

**Table 1: Regression analysis of siderophore production in *Pseudomonas monteilli* strain B8**

**,* significant at 1% and 5% level of significance respectively.

**Table 2: Regression analysis of growth in *Pseudomonas monteilli* strainB8**

| **Source** | **Degree of freedom** | **Sum of square** | **Mean of square** | **F-Value (Fischer’s test)** | **p-value Probability** |
| --- | --- | --- | --- | --- | --- |
| **Model** | 9 | 2.30 | 0.26 | 24.18 | 0.0002** |
| **X_1_-pH** | 1 | 0.13 | 0.13 | 12.73 | 0.0091** |
| **X_2_-Conc of Fe** | 1 | 0.49 | 0.49 | 46.10 | 0.0003** |
| **X_3_-Ammonium Nitrate** | 1 | 0.30 | 0.30 | 28.43 | 0.0011** |
| **X_1_X_2_** | 1 | 0.23 | 0.23 | 22.01 | 0.0022** |
| **X_2_X_3_** | 1 | 0.45 | 0.45 | 42.12 | 0.0003** |
| **X_1_X_3_** | 1 | 0.25 | 0.25 | 23.73 | 0.0018** |
| **X_1_^2^** | 1 | 0.019 | 0.019 | 1.77 | 0.2253 |
| **X_2_^2^** | 1 | 0.32 | 0.32 | 30.46 | 0.0009** |
| **X_3_^2^** | 1 | 0.100 | 0.100 | 9.45 | 0.0180* |
| **Residual Error** | 7 | 0.074 | 0.011 |  |  |
| **Lack of Fit** | 3 | 0.074 | 0.025 | 14516.52 | **<0.0001** (Significant)** |
| **Pure Error** | 4 | 6.800E-006 | 1.700E-006 |  |  |
| **Corrected Total** | 16 | 2.38 |  |  |  |
| **R^2^** |  | 0.9688 |  |  |  |
| **R^2^adj** |  | 0.9288 |  |  |  |

**,* significant at 1% and 5% level of significance respectively.
